# Can deep learning identify humans by automatically constructing a database with dental panoramic radiographs?

**DOI:** 10.1371/journal.pone.0312537

**Published:** 2024-10-24

**Authors:** Hye-Ran Choi, Thomhert Suprapto Siadari, Dong-Yub Ko, Jo-Eun Kim, Kyung-Hoe Huh, Won-Jin Yi, Sam-Sun Lee, Min-Suk Heo

**Affiliations:** 1 Department of Advanced General Dentistry, Inje University Sanggye Paik Hospital, Seoul, Korea; 2 Artificial Intelligence Research Center, Digital Dental Hub Incorporation, Seoul, Korea; 3 Department of Oral and Maxillofacial Radiology, School of Dentistry and Dental Research Institute, Seoul National University, Seoul, Korea; Ajman University, UNITED ARAB EMIRATES

## Abstract

The aim of this study was to propose a novel method to identify individuals by recognizing dentition change, along with human identification process using deep learning. Recent and past images of adults aged 20–49 years with more than two dental panoramic radiographs (DPRs) were assumed as postmortem (PM) and antemortem (AM) images, respectively. The dataset contained 1,029 paired PM-AM DPRs from 2000 to 2020. After constructing a database of AM dentition, the degree of similarity was calculated and sorted in descending order. The matched rank of AM identical to an unknown PM was measured by extracting candidate groups (CGs). The percentage of rank was calculated as the success rate, and similarity scores were compared based on imaging time intervals. The matched AM images were ranked in the CG with success rates of 83.2%, 72.1%, and 59.4% in the imaging time interval for extracting the top 20.0%, 10.0%, and 5.0%, respectively. The success rates depended on sex, and were higher for women than for men: the success rates for the extraction of the top 20.0%, 10.0%, and 5.0% were 97.2%, 81.1%, and 66.5%, respectively, for women and 71.3%, 64.0%, and 52.0%, respectively, for men. The similarity score differed significantly between groups based on the imaging time interval of 17.7 years. This study showed outstanding performance of convolutional neural network using dental panoramic radiographs in effectively reducing the size of AM CG in identifying humans.

## Introduction

Human identification is crucial when catastrophes cause large-scale deaths. After the 2011 east Japan earthquake and tsunami, approximately 10% of the total number of victims whose identities were confirmed were identified by dental examination. In the 2003 Daegu subway fire disaster in Korea, a similar pattern was observed. Disaster victim identification (DVI) by dentists has therefore garnered growing attention. Teeth and dental treatment types are useful in human identification because they are resistant to destruction by fire [1–3]. The use of teeth, one of the hard tissues in the human body, helps reduce the number of potential candidates and accurately confirms their identity.

With an increasingly aging society, identifying individuals by dental restorations is more likely because the number and complexity of dental restorations increase with age [4]. A previous study [5] reported that individuals who had received several complicated dental treatments were more easily identified than individuals who had not received or rarely received such treatments. A dental panoramic radiograph (DPR) is a reliable tool for human identification because it is regularly filmed and updated. DPRs, which contain most of the major dental information, are widely used in clinical practice and therefore have a high potential for clinical use.

In recent years, artificial intelligence (AI) has assumed an important role in the types of image processing that are cumbersome or time-consuming for the observer [6]. Object detection, image classification, and pattern recognition using deep learning have developed rapidly and are applied to medical diagnoses using dental imaging [7,8]. The convolutional neural network (CNN) method is prominent in the image domain and is particularly useful in finding patterns for image recognition [9], including radiological applications in general medical fields [9,10]. In particular, object detection has recently undergone remarkable development, and has achieved the establishment of an efficient model variously named “end-to-end learning” [11–13], “transfer learning” [14,15], “active learning” [16,17], and “explainable deep learning” [18,19]. Recently, explainable deep learning models have been increasingly introduced into biomedical image analysis to overcome the inherent black box problem.

AI has been most actively introduced in diagnostic imaging in the medical imaging area. Research on human identification conducted using deep neural networks began with detecting teeth and dental prostheses from DPRs [20–32]. Going further from the excellent research results on detection, the research trend of deep learning in the field of human identification is the comprehensive establishment and automation of human identification processes. Studies related to human identification that simply compared DPR images predominate. The mainstream themes of existing studies have been to find possible candidates based on the similarity of images or to measure similarities, based on various identifiers by extracting certain features or patterns from DPRs. Extracting features or performing comparisons on images could be intuitive and fast, but it may also be affected by several endogenous variables such as distortion, contrast, and resolution of DPRs [33].

To date, few studies have conducted comprehensive simulations of the human identification process using deep neural networks. To achieve the ultimate comprehensive human identification, an approach that employs database construction is essential; it takes one step forward from existing studies of human identification that are primarily based on traditional comparisons of the postmortem (PM) and antemortem (AM) images. When the database is constructed, various parameters can be extracted from the images, and developing a human identification methodology from diverse viewpoints would ultimately be possible. This study aimed to develop a novel automated system that can identify humans as a basis for DVI in the field of forensic science.

## Material and methods

DPR-pair dataset for constructing a database for the human identification model

The Institutional Review Board of Seoul National University Dental Hospital (SNUDH) approved this study (ERI20032) and waived the need for informed consent. The entire experiments including data acquisition were conducted under the relevant research regulations and guidelines. DPRs of 1,029 individuals were used to simulate the postulated human identification process. The dataset contained 2,058 DPRs comprising paired images of the recent and past DPRs for each individual. These images were randomly selected after removing identifiable patient information and were retrospectively reviewed from the picture archiving and communication system at SNUDH. All authors had not access to information that could identify individual participants during or after data collection. The radiographic images were obtained using panoramic radiographic machines OP-100 (Imaging Instrumentarium, Tuusula, Finland) and Rayscan Alpha P (Ray, Gyeonggi, Korea).

From January 2000 to November 2020, DPRs with information that include age and sex were collected for dental treatment or diagnosis. Recent and past DPRs of adults aged 20–49 years (465 men and 564 women) who had more than two DPRs were assumed as the PM and AM images, respectively. The timepoints at which the PM and AM DPRs were recorded were also collected as six digits in the format of two-digit year, two-digit month, and two-digit day. The age range was limited to 20–49 years to minimize the impact of dentition changes and image distortions over time, which could lead to false identifications. This range was chosen based on the exclusion of younger individuals with deciduous teeth and older individuals with higher rates of tooth loss, ensuring a more consistent and reliable analysis. Additionally, the age criteria used in the previous study [21] were maintained to ensure the validity of the model.

### Detection of natural teeth and dental treatment types using the pre-trained model

**Fig 1:**
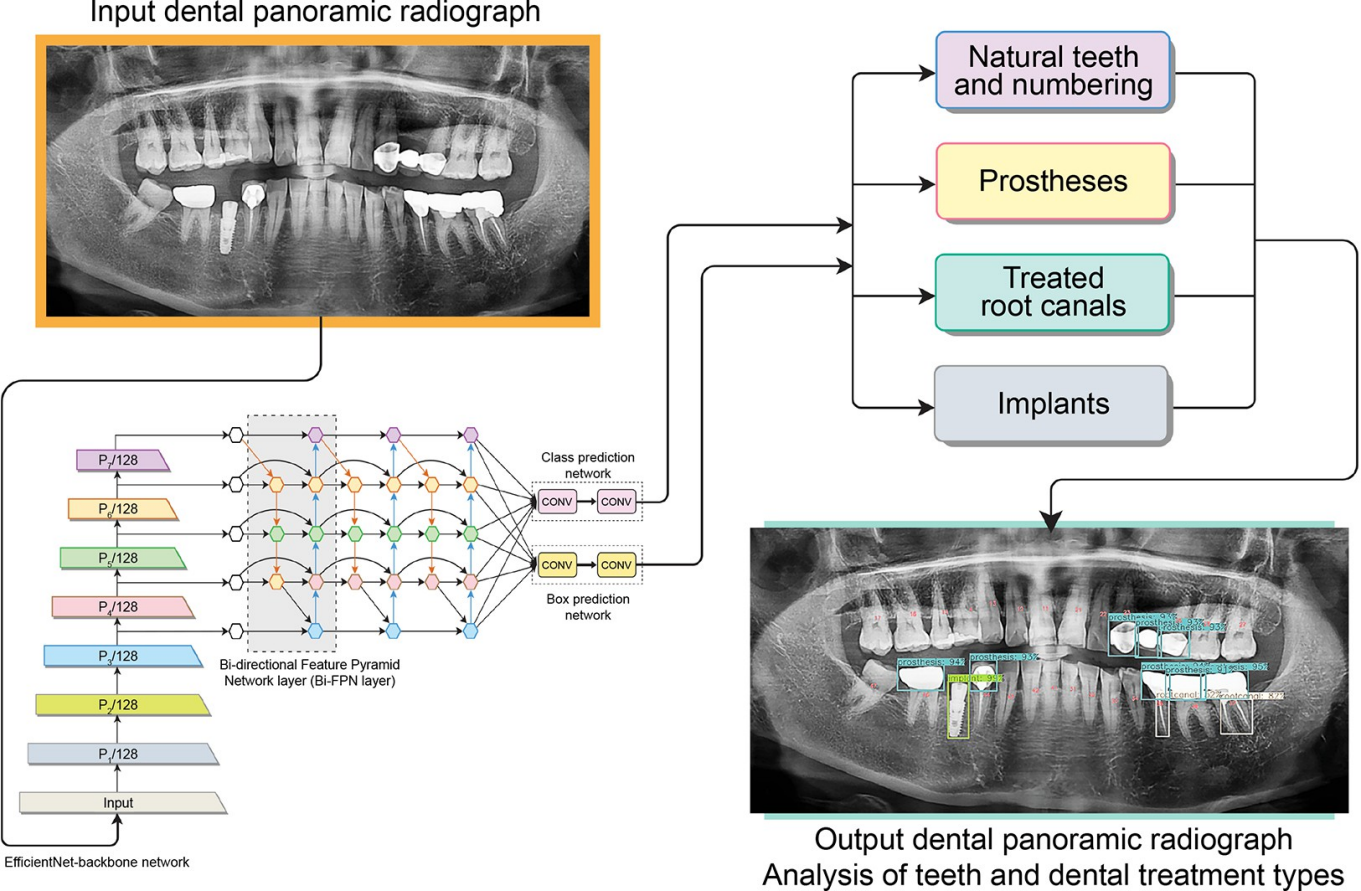
Deep neural network architecture for the detection of natural teeth and dental treatment types.

Natural teeth and dental treatment types were detected and the teeth numbers were identified using a pre-trained object detection network (Fig 1) which was a CNN modified by EfficientDet-D3 [21]. DPRs (n = 1,638) were collected in advance to construct this model. These DPRs were mutually exclusive from the existing 2,058 DPRs of 1,029 individuals. A fully web browser-based labeling system developed by Digital Dental Hub (DDH, Inc., Seoul, Korea) was used in the annotation task.

Table 1 presents the overall performance of the model, composition of the dataset and description of the subnetwork for each task in the network architecture. The performances were evaluated using the standard of average precision and average recall under the intersection over union (IoU) of 0.5. A true positive was considered only for prediction scores >50% and an IoU with a threshold value of 0.5.

**Table 1:** The performance of pre-trained network for object detection in dental panoramic radiographs.

Category^†^	The quantity of objects used in each stage of the network				Detection performance*	
	Training	Validation	Test	Total	Average precision	Average recall
**Natural teeth**	27,302	10,139	8,926	46,367	99.1%	99.6%
**Prostheses**	7,763	2,824	2,502	13,089	80.6%	84.3%
**Treated root canals**	2,170	813	684	3,667	81.2%	89.2%
**Implants**	643	212	157	1,012	96.8%	98.1%

*Average precision and recall values were calculated under a threshold value of the intersection over union of 0.5 for detection performance of natural teeth and dental treatment types.

^†^Natural teeth were in a pure state, for example, they had not undergone any treatment. Prostheses were defined as the parts that were directly or indirectly restored among the overall shape of teeth such as composite resin filling or dental inlay. Treated root canals referred to the part of roots that shows traces of root canal treatment. Implants were classified into an independent category distinct from prostheses. It was defined as a category of implants if an implant fixture was observed alone or an implant fixture with a crown was observed in a certain tooth location.

### Data post-processing

A database was developed by generating the dental information using the pre-trained model from each set of pairs of the PM and AM DPRs of 1,029 individuals. Detectable information that could be extracted from each DPR using deep neural networks varied substantially; therefore, it was simplified to focus on the main dental components. This dental information consisted of natural teeth, including tooth number, prostheses, treated root canals, and implants. Detection of dental pathologic conditions such as dental caries or periodontal lesion was not considered.

**Fig 2:**
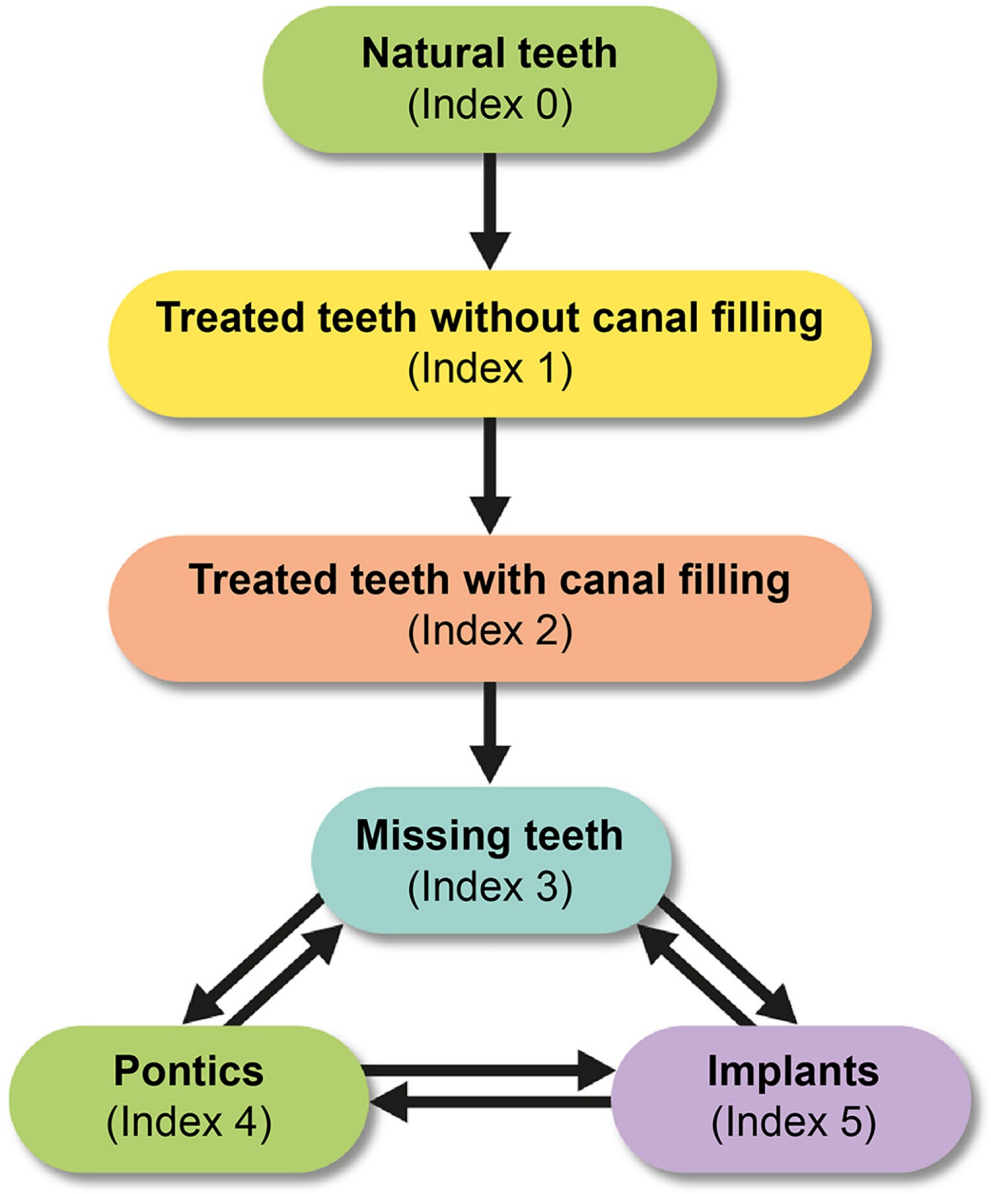
Index system based on the degree of dental treatment. The tooth status can be changed only in the direction of the arrow. The tooth status can be converted to each other in case of missing teeth, pontics, and implants.

Based on the state and direction of change of the individual’s teeth, the data regarding the detected natural teeth and dental treatment types were post-processed as objective values. As the teeth condition changed (Fig 2), the possible past status of the tooth condition for each tooth condition was determined. The summary of detailed past status was provided as supplementary data.

### Evaluation of the process of human identification

**Fig 3:**
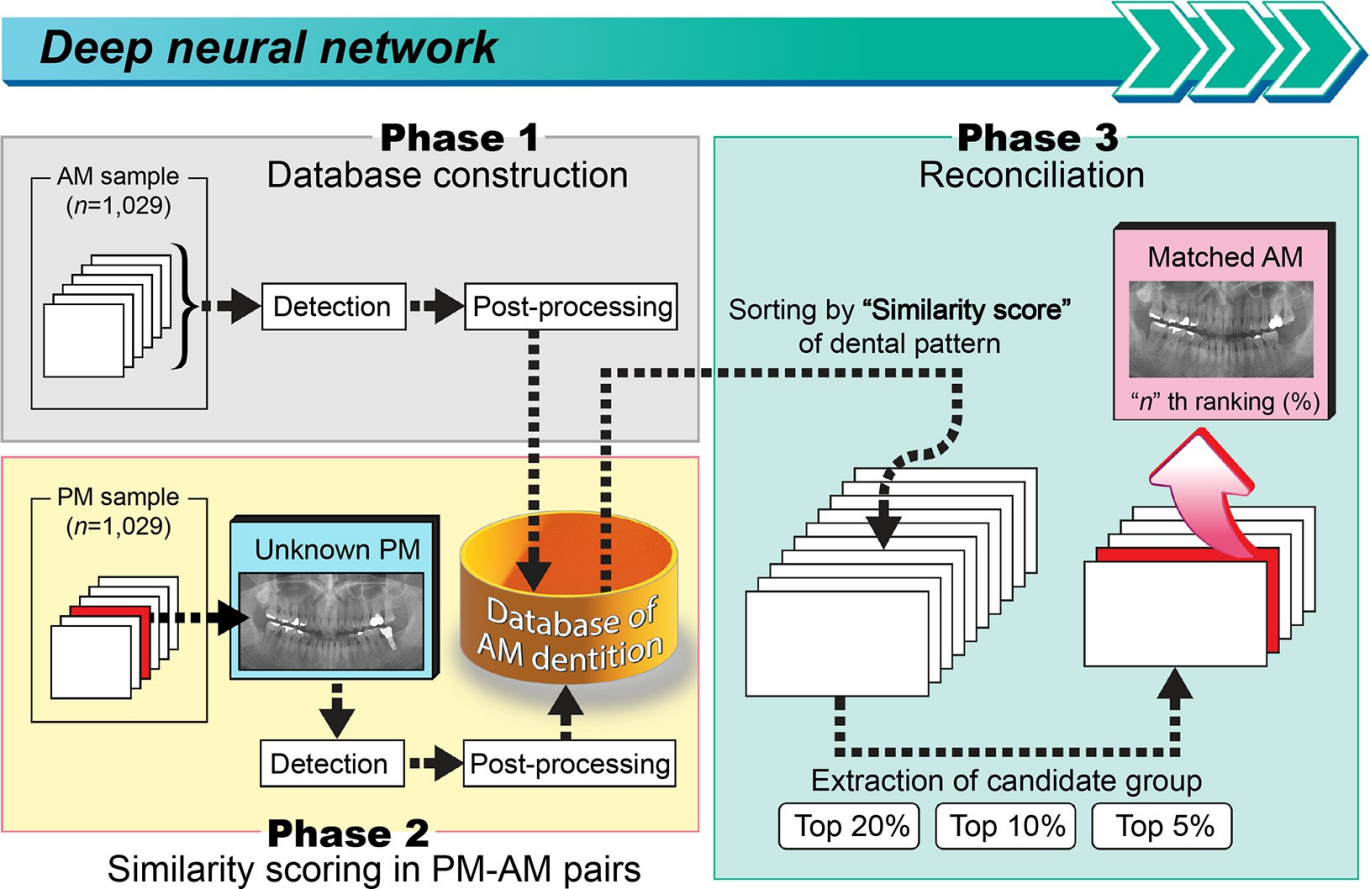
Scheme of the simulation of human identification using the automated human identification process, based on the dentition of an unknown postmortem (PM) individual. In Phase 1, a database of antemortem (AM) dentition was constructed. In Phase 2, similarity scores were calculated for each pair of PM-AM dental panoramic radiographs. In Phase 3, the scored similarities were sorted in descending order and extracted for the top 20.0%, 10.0%, and 5.0%. The matched rank was calculated as the success rate for the percentage.

To simulate the automatic process of human identification, the algorithm for human identification was implemented by deriving possible candidates from the AM database, assuming the situation of an unknown DPR in the PM database. Three phases were established (Fig 3).

In Phase 1, using the pre-trained model, all teeth numbers and therapeutic identifiers of 1,029 AM individuals were detected and post-processed. In Phase 2, the difference score was calculated to estimate the similarity score in the PM-AM pairs. This difference score was evaluated by comparing the dental condition at each position. A score of 0 points indicated that the position and the state were the same, and a higher score indicated that the distance difference between the tooth conditions increased. During this process, a relatively large score, the maximum of which was 10 points, indicated teeth for which the positions were unavailable in the past to increase the difference. A penalty of 10 points was imposed when an unexplainable discrepancy occurred. Table 2 summarizes the calculation methods of the difference and similarity scores. The difference score was subtracted from 1 to finally obtain the similarity score.

**Table 2:** The Student’s t-test analysis comparing the groups with shorter and longer imaging time intervals.

The imaging time intervals	N^†^	Similarity score
< 6,450 days	994	0.948±0.069*
≥ 6,450 days	35	0.915±0.091*

*p<0.05.

^†^Number of dental panoramic radiographs.

In Phase 3, similarity scores were calculated by 1:1 matching for dentition information of one anonymous DPR and all AM dentitions. After sorting the derived similarity scores in descending order, the top 20.0%, 10.0%, and 5.0% candidate groups (CGs) were extracted. The success rate was calculated after the rank position was checked to determine whether matched AMs existed in this group, as follows:

$$Success\;Rate\;(\%)=\left(\frac{Rank\;of\;correct\;AM\;DPR\;within\;the\;CG}{Total\;Number\;of\;DPR\;Pairs\;in\;the\;CG}\right)\times 100$$

The same procedure as described above was conducted within each sex group. All aforementioned processes were fully automated based on a deep neural network.

### Comparison of similarity scores among the groups, based on the imaging time interval

The imaging time interval was calculated as the number of days by subtracting the first date the DPR was taken from the most recent date the DPR was taken. The cut-off value was determined by using the regression method to analyze whether the similarity scores were statistically different, based on the interval between the PM and AM imaging times. The imaging time intervals, a sequence of consecutive numbers, were first divided into units of 100 days to set a discrete section, and dummy variables were generated. The value was set to 0 and 1 in cases when the interval between imaging times was lower or higher than the discrete value, respectively. The individual regression model was used to establish the relationship between each of the discrete values of this interval between the imaging times and the dependent variable, which is the similarity score. The minimum value of the imaging time interval was 1 day and the maximum value was 7,732 days. Therefore, 77 regression equations were derived because the dummy variables were introduced by dividing by 100 days, as follows:

$$Y_{i}=b_{0j}+b_{1j}X_{i}+e_{j}\;(i=1\ldots 1029,\;j=1\ldots 77)$$

where i and j are natural number that range from 1 to 1029 (i = 1…1029) and from 1 to 77 (j = 1…77), respectively. *Y_i_* is similarity scores from 1,029 paired PM-AM DPRs and *X_i_* is a dummy variable from the division into units of 100 days of the imaging time intervals.

The 77 derived regression coefficients were plotted to analyze for trend. The section in which the trend changed most rapidly was set as the cut-off value. Finally, the groups were divided, based on this cut-off value, and the Student’s t-test was conducted. SPSS software version 23.0 for Windows (IBM, Chicago, IL, USA) was used for the statistical analyses.

## Results

### Descriptive statistics

The mean age of the individuals was 35.5±15.3 years; the youngest and oldest individuals were 20 and 49, respectively. The average imaging time interval between the most recent and past DPR was 2,197.5±1,934.7 days. The minimum value was 1 day and the maximum value was 7,732 days.

### Analysis of the similarity scores between the imaging time interval groups

The plot of each derived regression coefficient is shown in S1 Fig. The section or imaging time interval between 6,400 and 6,500 days, in which the trend changed most rapidly, was chosen as the cut-off value. The regression coefficient showed the most rapid change from -0.22 to 0.15. Thus, this cut-off value was finally determined to be 6,450 days. Based on this cut-off value, the variables of the imaging time interval were divided into two groups. As shown in Table 2, the similarity score was significantly different between the two groups, based on the period between the date of the most recent DPR and that of the past DPR imaging at 17.7 years (p = 0.006).

### Performance of the human identification process

The matched AM was ranked in the CG with a success rate of 83.2% for the extraction of the top 20.0% CG. The target individual was, on average, in the top 20.0% CG with 83.2% probability. The success rates were 72.1% and 59.4% for the extraction of the top 10.0% and top 5.0%, respectively. The success rate differed, based on sex (S2 Table). The success rates for the extraction of the top 20.0%, 10.0%, and 5.0% were 71.3%, 64.0%, and 52.0%, respectively, for men and 97.2%, 81.1%, and 66.5%, respectively, for women. S3 Table presents the performance of the human identification process in a group with an imaging time interval of <6,450 days.

## Discussion

The aims of this study were to construct a database of individuals’ dentition with DPRs, to use a novel method based on deep neural networks to identify individuals by recognizing their dentition changes using a pre-trained object detection network, and to evaluate the feasibility of this novel method. We found that the developed method was useful in detecting individuals by their dentition.

To date, different models for human identification have been proposed. The latest study [34] developed an identification system using DPRs with the Visual Geometry Group 16-layer model showing the highest accuracy, and other similar studies [35,36] developed more sophisticated models to better extract features that improve matching performance. A previous study confirmed the decisive role that the sorting method has in human identification while considering similarity [37]. A database search based on the basic assumption regarding dentition changes and sorting using similarity calculation was useful in human identification. All processes, however, including database construction and sorting by similarity calculation, were conducted manually in this study.

The significance of this study is that, rather than comparing the images, the dental information from DPR images was converted into objective information to prevent errors that can be generated from analyses using simple image comparison in advance. An excellent model was developed for this information conversion. The superiority of the model in automatically detecting natural teeth and dental treatment types has already been acknowledged through a preliminary study [21]. This study considerably contributes to the whole process of human identification that considers PM and AM similarities automated through deep learning, which includes advanced automatic identification of natural teeth and dental treatment types and the construction of an individual database based on this information. During the entire identification process, 32 teeth were classified into six states to increase the diversity of teeth arrangement. A step was added to compress and derive a high-probability group from possible candidates into a group. We believe that assigning directionality to tooth change increased the probability of identification.

To summarize, the top three probabilities of the target human identification were 83.2%, 72.1%, and 59.4%, which were obtained by respectively extracting the top 20.0%, 10.0%, and 5.0% of observations from the calculated similarity scores. The scoring was iterated 1,029 times, and the probabilities were sorted in descending order (Fig 3). Therefore, every anonymous PM was paired with the AM DPRs for the similarity scoring. The success rate differed between men and women. The success rate of the top 20.0%, 10.0%, and 5.0% CGs was 71.3%, 64.0%, and 52.0%, respectively, among men and 97.2%, 81.1%, and 66.5%, respectively, among women. These favorable success rates, especially when extracting the top 20%, underscore the potential of DPRs in practically identifying victims, even when other identifying features are unavailable or have deteriorated.

A possible reason the overall success rate was higher among women is that the dental information was extracted more accurately from women than from men by deep learning. The more precise extraction of dental information from women could potentially be influenced by diverse factors such as sexual dimorphism in teeth, the higher demand for orthodontic treatment among women, gender differences in oral health maintenance, and variations in periodontal disease progression between sexes [38,39]. The findings of our study could lead to more tailored approaches in practical forensic investigations, where gender-specific characteristics are considered during the identification process, thereby improving overall accuracy.

The difference in the similarity scores between the groups was statistically significant when the imaging time interval was 17.7 years. If the interval between the imaging times was <18 years, the interpretation is that the similarity between the two DPRs is high. When determining the cut-off value, a key aspect of our approach was to segment the imaging time intervals into intuitively data-driven 100-day intervals using ’day dummy’ variables, followed by regression analysis to examine differences between the two groups. To achieve a more refined determination of the cut-off value, alternative statistical methods, such as nonlinear regression analysis, could be considered.

As dental treatments become more common, it is very unlikely for a person to visit a dental clinic for the first time in 18 years and to take a DPR. Thus, it serves as an objective and pragmatic basis for supporting the effectiveness of the human identification algorithm based on similarity in the present study. In conclusion, an appropriate interval of <18 years between the imaging times for human identification is imperative when using the individual database. This insight is crucial for forensic experts as it suggests that the likelihood of successful identification decreases as the imaging time interval increases, emphasizing the need for timely collection and comparison of dental records in DVI scenarios.

Calculating the rank revealed that the performance was lower when 5.0% of CGs were extracted than when 10.0% were extracted. In this study, equally ranked individuals were assigned the same rank, and the next individual was ranked by adding the number of people in the preceding rank. To improve the performance of this model, further consideration for the handling of equal score processing is necessary.

In this model, the similarity decreases when the model is applied to AM images showing no restorations. For example, the condition with the worst outcome was an AM image of an individual with 32 natural teeth (including four third molars) with no restorations. Subsequent extraction of a third molar and treatment on several teeth may have caused poor results because of lowered DPR similarity. In instances in which the similarity score is too low or a discrepancy occurs, completely removing the CG could also be considered. However, in this study, CGs were not removed, but penalties were given: a penalty of 10 points was given to greatly increase the difference in tooth change during the step for deriving possible CG.

In the DPRs in which the pre-trained model was used, several unclear cases occurred during the dental treatment type detection stage. For instance, a restoration treatment such as a gold inlay through indirect restoration could be damaged or lost over time, and if the individual receives a direct restoration in the following treatment, the shape of the restoration would appear differently on the DPR, often resulting in errors when read by the network. Even when studied by specialists, ambiguous cases are often difficult to distinguish as to whether the tooth was natural or had received a restoration treatment using a resin.

A follow-up study is warranted to divide the prosthetic part as an indirect or direct restoration and to use a large number of samples for learning so that the neural network may distinguish between these two situations. Furthermore, human identification was conducted using dental information detected via deep learning only without considering the estimated age and the pathologic condition such as dental caries or periodontal lesion. Adding these pathologic conditions and the estimated date of birth of an individual would further improve the performance of reducing the CG. Adding conditions other than teeth arrangement and invariable anatomical structures such as the mental foramen, maxillary sinus, and anterior nasal spine would further increase the success rate of human identification.

Another critical consideration when establishing a more elaborate human identification system is addressing the inherent issues in AI model development. To mitigate the risks of data bias and imbalance, it is crucial to implement data augmentation and balanced sampling techniques, while also incorporating bias detection algorithms to ensure fairness across the model. Overfitting can be prevented by utilizing cross-validation methodologies. Incorporating interpretable algorithms or tools enhances the transparency of the decision-making process. Ethical considerations should be continuously evaluated and refined, with transparency maintained throughout the modeling and data collection stages. Ongoing updates and iterative retraining of the model are vital for sustaining real-world performance, and it is crucial to rigorously monitor the model’s efficacy on real-world data. Finally, regular monitoring for performance degradation due to environmental changes, coupled with routine updates, is essential for ensuring the model’s long-term sustainability.

The establishment of individual database using DPRs has various advantages, in addition to its application in the dental and medical fields. AI can integrate heterogeneous data domains such as demographic and clinical data, image data, biomolecular data, and social network data [40,41]. When a big database is ultimately established using DPRs, the DPR database would be highly reliable identifiers that could represent individuals, as fingerprints do.

## Conclusion

This study demonstrated the feasibility of human identification regarding dentition change on DPRs by not only automatically detecting dental objects but also constructing a comprehensive database through an indexing system. The developed method enhances the accuracy of the identification process by surpassing traditional image comparison trends. This approach could effectively reduce the size of an AM CG to be reviewed, especially in DVI scenarios, while serving as a powerful tool for dental professionals and other stakeholders.

## Supporting information

S1 Fig Determination of the cut-off value of the imaging time intervals from the trend analysis of the regression coefficients. (PDF)

S1 Table Method used to calculate the similarity score. (PDF)

S2 Table Success rates of human identification in the entire imaging time interval. (PDF)

S3 Table Success rates of human identification for the imaging time interval of <6450 days. (PDF)
