# Supplementary material for: Can deep learning identify humans by automatically constructing a database with dental panoramic radiographs?
Source: PLoS One. 2024 Oct 24;19(10):e0312537. doi: 10.1371/journal.pone.0312537 (PMC11500890; doi:10.1371/journal.pone.0312537)
Supplement: S1 Fig — (PDF) [file pone.0312537.s001.pdf]

Supplementary Material

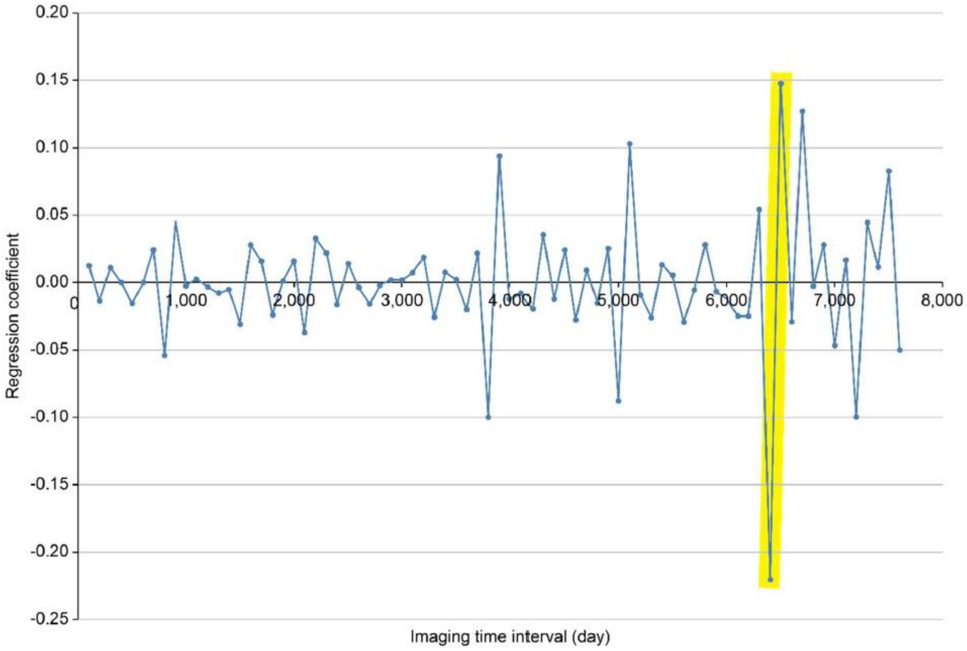

Figure S1. Determination of the cut-off value of the imaging time intervals from the trend analysis of the regression coefficients.
