# Supplementary material for: Can deep learning identify humans by automatically constructing a database with dental panoramic radiographs?
Source: PLoS One. 2024 Oct 24;19(10):e0312537. doi: 10.1371/journal.pone.0312537 (PMC11500890; doi:10.1371/journal.pone.0312537)
Supplement: S1 Table — (PDF) [file pone.0312537.s002.pdf]

Table S1. Method used to calculate the similarity score

Teeth status of Postmortem (PM) (index score)	Teeth status of Antemortem (AM) (index score)	Difference score*
Natural teeth (0)	Natural teeth (0)	(PM score – AM score)
Treated teeth without canal filling (1)	Natural teeth (0)	10 (Penalty score)
	Etc.	(PM score – AM score)
Treated teeth with canal filling (2)	Natural teeth (0)	10 (Penalty score)
	Treated teeth without canal filling (1)	
	Etc.	(PM code – AM code)
Missing teeth[†] (3)	Natural teeth (0)	10 (Penalty score)
Pontics (4)	Treated teeth without canal filling (1)	
Implants (5)	Treated teeth with canal filling (2)	
	Etc.	If AM tooth status was equal to PM tooth status, then score was 0. Otherwise, score was 1.

[*]Difference scores were summed up for all teeth positions and finally divided by 320, which represents the total number of teeth multiplied by the penalty score. The difference score was subtracted from 1 to obtain the similarity score.

[†]The states of missing teeth, pontics, and implants can be interchanged.
