# Supplementary material for: Can deep learning identify humans by automatically constructing a database with dental panoramic radiographs?
Source: PLoS One. 2024 Oct 24;19(10):e0312537. doi: 10.1371/journal.pone.0312537 (PMC11500890; doi:10.1371/journal.pone.0312537)
Supplement: S2 Table — (PDF) [file pone.0312537.s003.pdf]

Table S2. Success rates of human identification in the entire imaging time interval

The extraction rates of top candidate group	Success rate		
	Total	Men	Women
20.0%	83.2%	71.3%	97.2%
10.0%	72.1%	64.0%	81.1%
5.0%	59.4%	52.0%	66.5%
