# Supplementary material for: Can deep learning identify humans by automatically constructing a database with dental panoramic radiographs?
Source: PLoS One. 2024 Oct 24;19(10):e0312537. doi: 10.1371/journal.pone.0312537 (PMC11500890; doi:10.1371/journal.pone.0312537)
Supplement: S3 Table — (PDF) [file pone.0312537.s004.pdf]

Table S3. Success rates of human identification for the imaging time interval of <6450 days

The extraction rates of top candidate group	Success rate		
	Total	Men	Women
20.0%	84.0%	71.3%	97.8%
10.0%	72.7%	63.6%	81.8%
5.0%	59.4%	51.8%	66.9%
